# Lymphotoxin-beta receptor blockade reduces CXCL13 in lacrimal glands and improves corneal integrity in the NOD model of Sjögren's syndrome

**DOI:** 10.1186/ar3507

**Published:** 2011-11-01

**Authors:** Roy A Fava, Susan M Kennedy, Sheryl G Wood, Anne I Bolstad, Jadwiga Bienkowska, Adrian Papandile, John A Kelly, Clio P Mavragani, Margaret Gatumu, Kathrine Skarstein, Jeffrey L Browning

**Affiliations:** 1 Immunology Research Department, Department of Veterans Affairs Medical Center, 215 North Main Street, White River Junction, VT 05009, USA; 2 Department of Medicine, Dartmouth Medical School, 1 Rope Ferry Road, Hanover, NH 03755, USA; 3 Department of Clinical Dentistry, University of Bergen, Årstadvn. 17, N-5009 Bergen, Norway; 4 Department of Immunology, Biogen Idec, 14 Cambridge Center, Cambridge, MA 02142, USA; 5 Department of Experimental Physiology, School of Medicine, University of Athens, M. Asias 75, Goudi, 11527 Athens, Greece; 6 The Gade Institute, University of Bergen, P.O. Box 7800, Bergen 5020, Norway

**Keywords:** Lymphotoxin-beta, Sjogren's syndrome, chemokine, CXCL13, keratoconjuntivitis sicca, NOD mouse

## Abstract

**Introduction:**

In Sjögren's syndrome, keratoconjunctivitis sicca (dry eye) is associated with infiltration of lacrimal glands by leukocytes and consequent losses of tear-fluid production and the integrity of the ocular surface. We investigated the effect of blockade of the lymphotoxin-beta receptor (LTBR) pathway on lacrimal-gland pathology in the NOD mouse model of Sjögren's syndrome.

**Methods:**

Male NOD mice were treated for up to ten weeks with an antagonist, LTBR-Ig, or control mouse antibody MOPC-21. Extra-orbital lacrimal glands were analyzed by immunohistochemistry for high endothelial venules (HEV), by Affymetrix gene-array analysis and real-time PCR for differential gene expression, and by ELISA for CXCL13 protein. Leukocytes from lacrimal glands were analyzed by flow-cytometry. Tear-fluid secretion-rates were measured and the integrity of the ocular surface was scored using slit-lamp microscopy and fluorescein isothiocyanate (FITC) staining. The chemokine CXCL13 was measured by ELISA in sera from Sjögren's syndrome patients (*n *= 27) and healthy controls (*n *= 30). Statistical analysis was by the two-tailed, unpaired T-test, or the Mann-Whitney-test for ocular integrity scores.

**Results:**

LTBR blockade for eight weeks reduced B-cell accumulation (approximately 5-fold), eliminated HEV in lacrimal glands, and reduced the entry rate of lymphocytes into lacrimal glands. Affymetrix-chip analysis revealed numerous changes in mRNA expression due to LTBR blockade, including reduction of homeostatic chemokine expression. The reduction of *CXCL13, CCL21, CCL19 *mRNA and the HEV-associated gene *GLYCAM-1 *was confirmed by PCR analysis. CXCL13 protein increased with disease progression in lacrimal-gland homogenates, but after LTBR blockade for 8 weeks, CXCL13 was reduced approximately 6-fold to 8.4 pg/mg (+/- 2.7) from 51 pg/mg (+/-5.3) in lacrimal glands of 16 week old control mice. Mice given LTBR blockade exhibited an approximately two-fold greater tear-fluid secretion than control mice (*P *= 0.001), and had a significantly improved ocular surface integrity score (*P *= 0.005). The mean CXCL13 concentration in sera from Sjögren's patients (*n *= 27) was 170 pg/ml, compared to 92.0 pg/ml for sera from (*n *= 30) healthy controls (*P *= 0.01).

**Conclusions:**

Blockade of LTBR pathways may have therapeutic potential for treatment of Sjögren's syndrome.

## Introduction

Sjögren's syndrome is an autoimmune exocrinopathy affecting most secretory glands, but especially the salivary and lacrimal glands. As the disease progresses, leukocytes accumulate in salivary and lacrimal glands. This results in hypo-secretion of saliva and tear fluids causing xerostomia or dry mouth and keratoconjunctivitis sicca (KS) or dry eye, respectively. The infiltrating lymphocytes in salivary gland biopsies are often organized into tertiary lymphoid tissues (TLT) with segregated T- and B-cell zones and follicular dendritic cell (FDC) networks [1]. Some of the TLT are engaged in germinal center reactions, evidenced by expression of activation-induced cytidine deaminase (AID) [1,2], although one report indicates active germinal center reactions may be relatively rare [3]. Whether the immune reactions that occur within TLT exert harmful or beneficial effects is not yet clear. Experimental evidence exists for both possibilities, suggesting that the effects of immune reactions in TLT vary with organ and disease context [4].

The lymphotoxin-beta receptor (LTBR) pathway has been associated with the presence of TLT (or ectopic follicles) at sites of chronic inflammation in several autoimmune diseases [4,5]. LTBR directly controls several gene products that contribute to tertiary lymphoid tissue development, including homeostatic chemokines (CCL19, CCL21, CXCL13) and several proteins required for peripheral lymph node addressin (PNAd) assembly on high-endothelial venules [6,7]. Therefore, CXCL13 and the lymphotoxin-beta receptor pathway are considered essential to development of tertiary lymphoid tissues and might constitute a useful therapeutic target in certain diseases [4,7-9]. In minor salivary gland biopsies from patients with Sjogren's syndrome, lymphotoxin-beta was the fifth most differentially expressed gene, with expression approximately eight fold higher than in gland biopsies from healthy control subjects [10]. LTBR is expressed in epithelium of salivary glands in mouse embryos from day 16.5 onward; expression in lacrimal glands has not yet been formally documented [11]. Interestingly, CXCL13 also was one of only five genes expressed in >90% of the Sjogren's patient biopsies (but <10% of control biopsies) and CXCL13 expression has been localized to ectopic follicles in salivary glands in Sjogren's syndrome, making its expression in salivary glands a possible disease marker [1,12].

In murine models of the disease, as in humans, Sjögren's syndrome occurs both as a primary disease and as a secondary disease associated with autoimmune diseases such as lupus, scleroderma, diabetes and rheumatoid arthritis [13]. For example, the female NOD mouse that is often used to study the salivary gland aspects of Sjögren's syndrome also develops diabetes concurrent with salivary gland pathology. The salivary gland disease in female NOD mice is not dependent on the diabetes however [14]. Each disease derives from unique chromosomal regions (salivary gland disease versus diabetes) with one chromosomal region containing the genes that cause diabetes and a different chromosomal region encoding the salivary gland disease [15]. The two regions have been physically separated and when one region was bred into an autoimmune resistant strain, it resulted in a transgenic mouse with Sjögren's-like salivary gland disease but without pancreatitis or diabetes [15]. In this regard, NOD mice arguably may be viewed as a model of primary Sjögren's syndrome [15]. While salivary gland pathology is prominent in female NOD mice, their lacrimal glands remain virtually unaffected. Therefore, female mice cannot be used as a model of the dry-eye disease, or keratoconjunctivitis sicca (KS), of Sjögren's syndrome,.

Less is understood about lacrimal gland pathology in Sjögren's syndrome than salivary gland pathology, largely due to the difficulty of obtaining biopsies of lacrimal glands compared to salivary glands. However, it is known that large leukocyte infiltrates form in lacrimal glands of patients with Sjögren's syndrome. Very similar leukocyte infiltrates develop in the lacrimal glands of male NOD mice used in this study, with coincident losses in gland secretory function [16-18]. Because of this, male NOD mice provide a useful model of dry-eye disease or KS. Recently, the male NOD mouse model was thoroughly characterized to illustrate fully its potential as a model of KS [19,20]. In contrast with female mice, male NOD mice rarely develop salivary gland disease or diabetes (approximately 10%), however very large leukocyte infiltrates spontaneously develop in their lacrimal glands at an early age, offering an ideal and practical model with which to investigate possible therapies for the KS component of Sjögren's syndrome [21,22].

Leukocyte infiltrates develop in essentially 100% of the lacrimal glands of male NOD mice starting as small, perivascular infiltrates first observed at about 5 weeks of age. By approximately 12 weeks of age, very large leukocyte infiltrates are formed along with high endothelial venules (HEV), that can be identified by immunoreactivity with the monoclonal antibody MECA-79 that recognizes PNAd [22]. An earlier study indicated that the HEV in diseased lacrimal glands capture leukocytes from the circulation and therefore HEV might be a very useful therapeutic target in Sjögren's syndrome [22]. Since the formation and maintenance of functional HEV in secondary lymphoid organs of mice is regulated by the LTBR axis [6], as well as in TLT in experimental disease models [7,8,23], LTBR is a novel therapeutic target in Sjögren's syndrome.

In this study, we tested the chimeric antagonist LTBR-Ig, as a possible long-term therapeutic reagent (8-10 week treatments) for KS in Sjogren's syndrome. Blockade of the LTBR-pathway for 8 weeks reduced the size of lymphocyte aggregates, blunted homeostatic chemokine expression (CXCL13 and CCL19) by approximately 2- to 5- fold and reduced by up to 30-fold the expression of HEV-related genes (GLYCAM-1 and carbohydrate (N-acetylglucosamine 6-O) sulfotransferase 4 (Chst4)) in lacrimal glands. Antagonism of the LTBR-pathway thus undermined HEV development and function, dramatically reducing the size of leukocyte infiltrates in lacrimal glands, and mediated a partial protection from losses in the secretion of tear-fluids and the integrity of the ocular surface.

## Materials and methods

### Chemicals and antibodies

Pilocarpine was purchased from Sigma (St. Louis, MO, USA). Collagenase D used for tissue digestion for flow cytometry (FACS) analysis was purchased from Roche Applied Sciences (Indianapolis, IN, USA). A 15-minute digestion at room temperature with this type of collagenase did not affect the ability to detect relevant antigens on lymphocytes by flow-cytometry (B220, CD3, CD4, CD11c, CD23, CD21, CD45) (data not shown).

Conjugated antibodies for FACS analyses were purchased from BD Pharmingen (San Diego, CA, USA). In some experiments, a custom conjugated cocktail of antibodies (Jeff Browning, Biogen/Idec, Cambridge, MA, USA) was used for ten-color B-cell, T-cell and DC phenotyping.

MECA-79 (rat IgM) for PNAd staining in paraffin and frozen tissues was from BD Pharmingen and was developed with secondary horseradish peroxidase (HRP)-conjugate (goat-anti-rat IgM) from Jackson ImmunoResearch Laboratories (West Grove, PA, USA). Anti-B220 and anti-CD3 for paraffin staining (rat anti-mouse B220 Cat# 1258G; rat anti-human/mouse CD3 Cat# MCA1477) were purchased from Serotec (Raleigh, NC, USA) and was developed with Vector ABC Horseradish Peroxidase Kit.

### Patient sera

Sera from 27 patients with primary Sjögren's syndrome who were being followed in the Rheumatology Department of General Hospital of Athens, Athens, Greece and 30 healthy controls were analyzed for CXCL13 protein levels. The diagnosis of primary Sjögren's syndrome was based on previously published criteria [24]. All patients and controls provided informed consent prior to participation to the study. Determination of CXCL13 protein levels was performed with a solid phase ELISA kit according to the manufacturer's instructions (Human CXCL13/BLC/BCA-1 Quantikine ELISA Kit; R&D Systems, Minneapolis, MN, USA). The mean age of Sjögren's syndrome patients was 57.17 +/- 10.10 years, and for controls was 57.55 +/- 21.17 years. There was one man in each group.

### Mice and treatments

The local VA Medical Center Institutional Animal Care and Use Committee approved all experiments and experimental procedures performed in this study and all mice were housed in an Association for Assessment and Accreditation of Laboratory Animal Care accredited facility. All male and female NOD mice were purchased from Taconic (Germantown, NY, USA). Male mice were received when they were 5 to 6 weeks old and female mice when they were 7 weeks old. The systemic antagonist, mLTBR-mIgG1 (LTBR-Ig) and a control murine monoclonal IgG1 antibody MOPC-21 were prepared at Biogen/Idec and stored at -80°C until used. In all experiments intra-peritoneal injections of 100 μg of either substance were performed once a week. Unless indicated, all data shown are representative of at least three independent experiments, executed over a period of four years with NOD mice from one vendor. The dose and injection regimen for LTBR-Ig is known to elicit maximal biologic effects in other autoimmune animal models [6,25] and was confirmed in experiments with NOD mice [26]. Efficacy was confirmed in male NOD mice by the depletion of splenic marginal zone B-cells after as long as 10 weeks of injections of LTBR-Ig (data not shown).

### Histochemistry, immuno-histochemistry and morphometry

Organs were fixed with 10% formalin and embedded in paraffin by standard methods or were embedded without fixation in Optimal Cutting Temperature (OCT) embedding compound for frozen sections. To quantify the PNAd-stained HEV content of lacrimal glands, a series of paraffin embedded tissue sections was prepared from eight lacrimal glands (four mice) per treatment group and sections were stained with MECA-79 monoclonal and anti-rat-IgM-HRP and slides developed with diamino-benzidine (DAB) as substrate. Randomly chosen fields (*n *= 20) were photographed with a Zeiss bright-field microscope and for each microscopic field, the area stained with DAB (the HEV area) and the total area of the gland was measured with NIH-Image software (5 fields/mouse). The mean and standard deviation of the percent of the area of HEV relative to the total area was then computed. For frozen sections, lacrimal glands were bisected longitudinally to optimize OCT infiltration and adhesion to slides (Fisher PLUS). Organs were kept approximately 5 minutes at room temperature in OCT compound, then frozen on dry ice and stored at -20°C until sectioned. Sections of 10 μm thickness were air dried for 30 minutes, fixed for 15 seconds with -20°C acetone, and then air dried overnight before immunostaining. Immunofluorescent microscopy was performed with a Nikon Eclipse 80i with a monochrome Retiga EXi CCD camera to enhance sensitivity and pseudocolor image capture and analysis with Nikon Elements software. Custom, narrow band filters for Texas red and Cy5 were used for overlay of multi-color images. Quantitation of PNAd staining was performed with a Zeiss Axioskop brightfield scope with SPOT software for image capture and NIH Image software for analysis.

### Differential gene expression analysis and quantitiation

Mice were euthanized by carbon dioxide inhalation. With the aid of a Zeiss surgical microscope cervical lymph nodes were carefully dissected away from submandibular glands and the combined cervical nodes and combined submandibular/salivary/parotid glands snap frozen on dry ice. Each lacrimal gland was dissected; both glands were combined from each of four mice and snap frozen (four biological replicates). Where noted, single lacrimal glands were snap-frozen for PCR while the contra-lateral gland was used for FACS analysis. Total mRNA was isolated from each sample with Trizol according to manufacturer instructions and DNase treated (DNA-free kit, Ambion, Austin, TX, USA), quantitated using the Nanodrop (Nanodrop Technologies, Wilmington, DE, USA) and the quality was assessed using the Agilent 2100 Bioanalyzer (Agilent Technologies, Palo Alto, CA, USA). Reverse transcription to prepare cDNA was performed using the M-MLV system, Invitrogen, Frederick, MD, USA.

Preliminary real-time PCR analyses (CXCL13, CCL21, CCL19, GAPDH) were performed with primer sets and SYBR Green master mix from SA Biosciences (Frederick, MD, USA), using a Bio Rad iQ5 iCycler with associated software (data not shown). Quantification was computed relative to target gene expression levels in healthy lacrimal gland from 20 week old female NOD mice. Data obtained by multiplex real time PCR analysis, shown in Results, was performed using Taqman primer sets (CXCL9, CXCL12, CXCL13, CCL19, CCL20, CCL21a, Chst2, Chst4, GlyCAM-1, VCAM, MAdCAM) from ABI using the Applied Biosystems 7900HT PCR System (Carlsbad, CA, USA). Target gene quantification (*n *= 7) was computed relative to the mean (*n *= 4) gene expression levels in healthy lacrimal glands from 20 week old female NOD mice.

Gene transcription profiling was carried out with Mouse Genome 430 2.0 arrays. All data processing and analysis were done using R and BioConductor packages [27]. Probe intensities in the whole set were normalized using the GCRMA (GC-corrected Robust Multi-Array Analysis) method. Four replicate samples were available for each tissue type, time point in the disease course and treatment versus control. First, genes present in at least two samples belonging to each group were identified. Second, gene expression values were estimated in each group using linear models as implemented by the limma package. We have identified differentially expressed genes between two sample groups using linear models (limma package), t-statistic, and Bayesian log-odds posterior probabilities. Genes with fold changes greater than 2 and Bayesian posterior probabilities less then 0.05 were selected as significantly differentially expressed between the groups. The data obtained for each Affymetrix array can be found at Accession Number: [GEO:GSE32681][28].

### CXCL13 ELISA of lacrimal-gland homogenates

Mouse organs collected for homogenization were quickly weighed and then snap frozen with dry ice and stored at -80 μC until used. Homogenates were prepared by thawing the tissue (approximately 10 to 20 mg) in 300 μl of ice-cold RIPA (radio-immuno-precipitation assay) buffer containing a cocktail of protease inhibitors and then homogenizing on ice for at least 60 seconds with a Polytron generator. The homogenates were centrifuged for 5 minutes at 13,000 RPM in a bench-top microfuge, the clarified supernatant solution collected, the total volume recorded and aliquots frozen for ELISA analysis. Mouse CXCL13 ELISA was performed using R&D Systems Quantikine kit after first determining that the presence of 50 μl radioimmuno-precipitation assay (RIPA) buffer did not significantly alter the ELISA assay. The volume of RIPA-buffer for homogenization was empirically determined to allow analysis of CXCL13 extracted from lymph nodes or lacrimal glands with 50 μl or less of homogenate (that is, 5 to 70 pg/mg tissue of CXCL13 in 50 μl of homogenate). The content of CXCL13 protein was determined for each individual lacrimal gland from groups of mice treated with LTBR-Ig from 8 to 16 weeks of age (*n *= 6) or untreated mice (*n *= 4), and the mean and standard deviation determined.

### CFSE-labeled lymphocyte uptake by lymph nodes and lacrimal glands

Carboxy-fluorescein-succinimidyl ester (CFSE) -labeled lymphocytes were prepared from cells pooled from 6 spleens and 12 cervical lymph nodes isolated from 6-week-old male donor NOD mice on the day of injection (CFSE-Cell Trace kit from Invitrogen). To enrich the CSFE-labeled cells for naïve cells (CD62L positive), the NOD donor mice were first injected with 150 μg of anti-CD40-ligand monoclonal antibody (MR-1), 5 days before isolation of spleens and lymph node cells for CFSE-fluorescence-labeling. MR-1 antibody depletes activated lymphocytes and approximately 40% of the CFSE-labeled donor lymphocytes used expressed CD62-L (data not shown). The ratio of T and B cells in the input cells was approximately 2:1. To label cells with CFSE, the pooled cells were incubated for 14 minutes at 37 μC with CFSE at 100 nM in 20 ml of Ca^++ ^and Mg^++^-free Hank's balanced salt solution, washed and suspended at 150 × 10^6^/ml. Recipient mice (five per treatment group) were given intravenous injections of 30 × 10^6 ^CFSE-labeled cells. Preliminary experiments indicated 20 hours was the minimum time required to reliably quantify CFSE-cells in lacrimal-gland infiltrates by flow-cytometry (data not shown).

Twenty hours after intravenous injection of CFSE-labeled cells, each pair of lacrimal glands was minced with micro-scissors 120 times, crushed between frosted-glass microscope slides, and the dispersed cells filtered through Falcon 70 μm mesh filters, washed and re-suspended in FACS-buffer. Lymph nodes were not minced. The isolated cells were stained with a multicolor antibody cocktail containing anti-CD45, anti-CD3, anti-CD4, anti-B220 and anti-CD62L and analyzed on a BD FACS Canto flow-cytometer. The total yield of leukocytes isolated from lacrimal glands was determined by trypan blue stain and counting by hemocytometer. This experiment was performed twice.

To calculate the uptake rate of circulating CFSE-labeled cells into lacrimal glands or nodes, 5 × 10^5 ^events were collected for the combined pair of lacrimal glands from each mouse (*n *= 5 mice of each treatment group). Initial gating was on CD45 positive cells to distinguish leukocytes from epithelial cells, nuclei and other debris in the homogenized glands. Note that CFSE cells entering lacrimal glands would be immediately diluted to a greater extent by the resident lymphocytes present in the glands of MOPC-21-treated mice than LTBR-Ig-treated mice; that is, there were always 3- to 4-fold more lymphocytes in glands of MOPC-21-treated mice than LTBR-Ig-treated mice. Specifically, for the experiment shown in the Results section there were 4.33 times more lymphocytes in the lacrimal glands of the MOPC-21-treated mice than in the LTBR-Ig-treated mice. The uptake rate per hour of CFSE-labeled cells into lacrimal glands or nodes was calculated and plotted per 10^6 ^CD45-positive cells.

### Determination of tear-flow rate

Tear-flow rates were determined both without, and with pharmacologic stimulation by intraperitoneal injection of pilocarpine, an agonist of parasympathetic nerve-pathways. Specifically, the method used for measuring the unstimulated tear-flow rate was a modification of a published procedure [29]. In brief, mice were anesthetized with isofluorane, and before beginning the measurement the end of a Zone-Quick phenol-impregnated thread was placed under the lower eyelid near the medial canthus for 10 seconds to absorb any previously secreted tear-fluid. The measurement was then begun by insertion of a second fresh Zone-Quick thread under the eyelid, which absorbed the tear-fluid secreted for 30 seconds. The length of wetted thread was measured. The means and standard deviations were determined and plotted, and two-tailed T-test performed.

To measure the pilocarpine-stimulated tear flow-rate, mice were weighed and injected with 0.5 mg/kg pilocarpine and anesthetized throughout the procedure by inhalation of isofluorane. Pilocarpine reaches a peak concentration in the circulation within 5 minutes post-injection that is maintained for about 10 minutes, declining afterward as the drug is metabolized. The tear-fluid that had accumulated under the eyelid during the first 5 minutes after injection of pilocarpine was first removed, and then the quantification of tear-flow was begun by insertion of a fresh Zone-Quick thread under the eyelid. Periodically during the 10-minute collection period, after approximately 10 mm of thread was wetted, the tear-saturated threads were removed and a fresh thread inserted. The total length of wetted thread was recorded for each eye and the mean of the lengths and standard deviations were calculated. The data shown are representative of three experiments.

### Scoring the integrity of ocular surface by slit-lamp microscopy

The integrity of the ocular surface was determined after FITC-staining, by examination of the eyes through a slit-lamp microscope (adapted for mice) using illumination with a Cobalt Blue filter. This adapted clinical method has been used previously for mice [30]. After lightly anesthetizing a mouse with isofluorane, 5 μl of a 0.03% solution of FITC in sterile PBS was placed onto the surface of the eye, and the lids manually opened and closed once. The mouse was then allowed to awaken for 2 minutes to naturally blink, then anesthetized again and the eye examined thoroughly (approximately 5 minutes) with Cobalt-Blue illumination with the mouse resting on a stage of the slit-lamp microscope. While under anesthesia, the eye of the mouse moves around slowly during the examination; therefore, the entire surface of the eye can be examined and given a score, based on a scale of 0 (no lesions) to 4 (see Additional file 6 for examples). The cornea was defined as the most central area above the pupil (limbus to limbus) and the conjunctiva as the remaining surface of the eye. This experiment was performed 3 times, with the observer blinded to treatment groups and 35 mice were examined overall. The score for each eye is displayed in a scatter-plot for a representative experiment, along with a mean standard deviation. Statistical analysis was by Mann-Whitney for non-parametric data sets.

## Results

### LTBR blockade reduces lymphocyte infiltration of lacrimal glands

**Figure 1:**
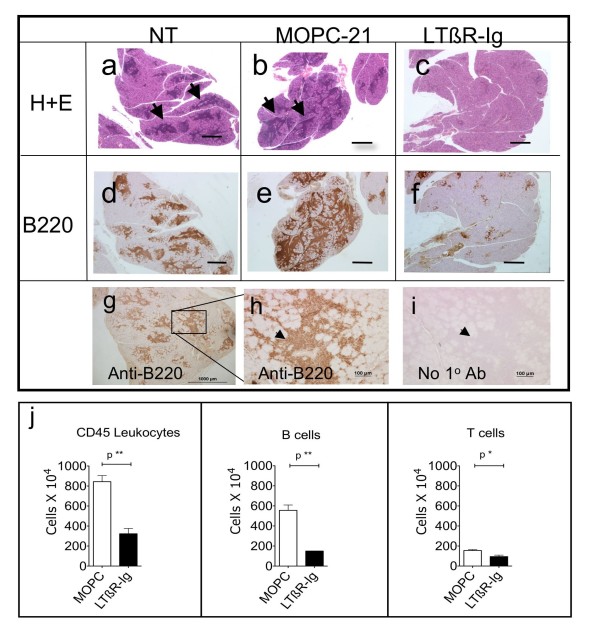
**Number of leukocytes in lacrimal glands is reduced by treatment with LTBR-Ig**. Representative photomicrographs of hematoxylin and eosin stained paraffin tissue-sections **(a to c) **and tissue-sections stained with anti-B220 and immuno-peroxidase (dark brown) **(d to h) **from NOD mice that were not treated (NT) or were treated from 8 to 16 weeks of age with MOPC-21 control antibody or with LTBR-Ig. Arrows (a,b) denote aggregations of leukocyte nuclei (dark blue). Bar equals 100 micron. Specificity control for anti-B220 staining of a section from a untreated mouse lacrimal gland (g); the area of the inset box in (g) is shown at higher magnification in (h) or an adjacent section stained without the primary antibody **(i)**; arrowhead marks same location in **(h,i)**. FACS analysis of leukocytes isolated from lacrimal glands of mice treated with MOPC-21 control antibody (*n *= 8 glands) or LTBR-Ig (*n *= 8 glands) **(j)**. Mean and standard deviation of total CD45 positive leukocytes, total number of B220 positive cells (B-cells) and of CD3 positive cells (T-cells) are indicated for each treatment type. This experiment was repeated four times. Statistical significance was calculated by two-tailed T-test. FACS, flow cytometry; LTBR-Ig, lymphotoxin-beta receptor-mouse immunoglobulin (Fc), NOD, non-obese diabetic.

Eight week-old male NOD mice were treated for 8 weeks with a systemic LTBR-axis antagonist, LTBR-Ig. Representative examples of leukocyte infiltrates in lacrimal glands of 16 week-old mice are shown in the very low magnification photomicrographs of tissue sections of whole glands (H+E stain) in Figure 1a-c, and a typical infiltrate is shown at much higher magnification in Additional File 1a. The leukocytes are tightly clustered and are visible in Figure 1a and 1b, as areas of dark-blue stained nuclei (examples marked by arrows). Compared to untreated mice and control MOPC-21 treated mice the clusters of leukocytes were greatly reduced in size and number by treatment of mice for 8 weeks with LTBR-Ig, as illustrated in Figure 1c. Massive leukocyte aggregations were present in the lacrimal glands of the oldest mice we examined (one year old), one example of which is shown in Additional File 1b. It was of interest that in one year-old mice (*n *= 4), the leukocyte aggregates after immunofluoresence staining for B-cells (blue), T-cells (red) and FDC (green), were often seen to be well organized, with well defined T-cell and B-cell zones. These hallmarks of tertiary lymphoid tissue formation (TLT) were observed in less than 10% of lacrimal-gland infiltrates of the mice aged 8 to 20 weeks that were used in this study.

The majority of lymphocytes present in lacrimal glands of mice 16 weeks of age were B-cells. After immunostaining with anti-B220 and detection by deposition of insoluble DAB substrate, B-cells were visible as massive clusters of dark brown stained cells, shown in Figure 1d, e. Specificity of the immunostaining pattern for B220 was confirmed by omitting primary antibody, shown in Figure 1g to 1i. As seen in Figure 1f, the aggregates of B-cells present in lacrimal glands were much smaller after treatment with LTBR-Ig. T-cells were also present in the lymphocyte aggregates (approximately 20% of leukocytes) but they were rarely found clustered together (data not shown). Staining for anti-CD3 by immunoperoxidase in paraffin sections, as well as immunofluorescence staining in frozen sections (data not shown) revealed that T-cells in lacrimal glands were most often observed as scattered, individual T-cells or occasionally were clustered in small groups of cells (<10 cells). This distribution of T-cells differs from that observed in the submandibular glands of female NOD mice of the same age, in which were found larger and more tightly grouped T-cells typical of organized TLT [26]. Interestingly, in the lacrimal glands of 1 year old male NOD mice (*n *= 4), distinctly segregated T-cell and B-cell areas were frequently observed, suggesting that more classical tertiary lymphoid follicles eventually do form in the lacrimal glands, as illustrated in Additional File 1b.

To quantify changes in the composition of the leukocyte infiltrates present in lacrimal glands after an 8-week treatment with LTBR-Ig, leucocytes were isolated from lacrimal glands and subjected to multicolor FACS analysis. As shown in Figure 1j, treatment with LTBR-Ig reduced the number of CD45 positive cells (*n *= 8 glands) by 3.2-fold compared to controls, specifically from approximately 8 × 10^6 ^per gland to approximately 3 × 10^6 ^per gland, respectively. A preferential reduction in the number of B-cells by LTBR-Ig treatment was observed, with a 4.8-fold reduction of B-cells (B220 positive) compared to only a 2-fold reduction of T-cells (CD3 positive), shown in Figure 1j. This result suggested that a large part of the mechanism(s) underlying the effect of LTBR-Ig antagonism on lymphocyte accumulation in lacrimal glands might be B-cell specific.

Three treatment regimens were used in our study, an early, preventative or prophylactic regimen (8 to 16 weeks of age), and two delayed or therapeutic regimens (10 to 20 weeks and 14 to approximately 22 weeks of age). All treatment regimens caused similar reductions (approximately 3-fold) in the total number of leukocytes in lacrimal glands of mice treated with LTBR-Ig, and particularly of B-cells, as shown for the prophylactic (8 to 16 weeks) regimen, in Figure 1 and for the therapeutic treatment regimen (14 to 22 weeks) in Additional File 3a.

### FACS analysis of leukocytes in lacrimal infiltrates, blood and spleen

To gain more insight into the abundance of various cell types present in lacrimal glands and the effect of LTBR-Ig treatment, we subjected leukocytes from lacrimal glands, spleen and blood to additional FACS analyses in mice 20 weeks of age, after a 10-week treatment with either or LTBR-Ig or the MOPC-21 control protein.

**Figure 2:**
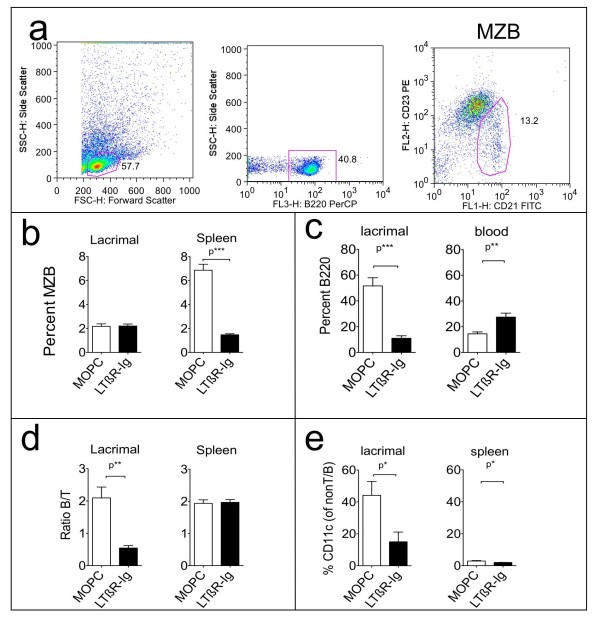
**FACS analysis of leukocytes in lacrimal glands, spleen and blood**. FACS analysis was performed on leukocytes from lacrimal-glands, spleen and in peripheral blood taken from NOD mice after treatment from 10 to 20 weeks with either MOPC-21 control protein (*n *= 7 mice) or LTBR-Ig (*n *= 8 mice), as indicated. Gating for marginal zone B-cells or MZB (B220+/CD23-int/CD21-hi) is depicted for spleen cells **(a) **with the MZB region indicated (red circle, third dot-plot). Treatment with LTBR-Ig reduced MZB in spleen but not lacrimal glands (b), reduced the percent of B-cells approximately 4-fold, whereas the percentage of B-cells in peripheral blood increased **(c)**, reduced the B-cell to T-cell ratio in lacrimal glands **(d) **and reduced the percentage of CD11c cells among non-T non-B-cells **(e)**. Similar experiments (treatment 8 - 16 weeks) were performed at least four times. LTBR-Ig; lymphotoxin-beta receptor-mouse immunoglobulin (Fc) chimeric inhibitor; MZB, marginal zone B-cell; NOD, non-obese diabetic.

Since others had reported an association of marginal zone B-cells with sialadenitis in a BAFF transgenic mouse model, we carefully examined this B-cell subset first [31,32]. The marginal zone B-cell (MZB) subset (B220-hi/CD21-hi/CD23-lo) was examined by gating on B220 and displaying CD21 and CD23 in a dot plot, as illustrated for splenocytes in Figure 2a. In the NOD mouse lacrimal glands, MZB were rare compared to spleen; a comparison of representative FACS dot-plots of splenic MZB and lacrimal gland MZB is shown in Additional File 3. As shown in Figure 2b, the mean percentage of MZB in lacrimal glands was considerably lower than that in spleen; and the majority of B-cells in lacrimal glands were conventional follicular B-cells (approximately 80%) as shown in a representative example in Additional File 3. As expected, in spleen the percentage of MZB was reduced by LTBR-Ig treatment, but the abundance of MZB was not affected in lacrimal glands, shown in Figure 2b. The reduction of MZB in spleen is a hallmark of LTBR-Ig antagonism [33,34] and confirmed that the LTBR-Ig chimeric protein antagonist had remained biologically active for the 10 weeks of administration. B220 positive cells that also expressed CD5 (putative B1-cell subset) constituted <2% of the B220 positive cells (data not shown).

The percentage of B-cells among all leukocytes isolated from the lacrimal glands was reduced about 5-fold to 11% (+/- 2.0) when mice were treated with LTBR-Ig, compared to 52% (+/- 6.2) for mice treated with MOPC-21 control protein, as shown in Figure 2c (*P *= 0.001). The opposite trend was found in the peripheral blood of the mice treated with LTBR-Ig, where the percentage of B-cells was higher compared to controls, shown in Figure 2c. Treatment with LTBR-Ig altered the ratio of B-cells to T-cells in the lacrimal glands approximately 2-fold (*p *= 0.01), but did not change the ratio in the spleen, as shown in Figure 2d.

**Figure 3:**
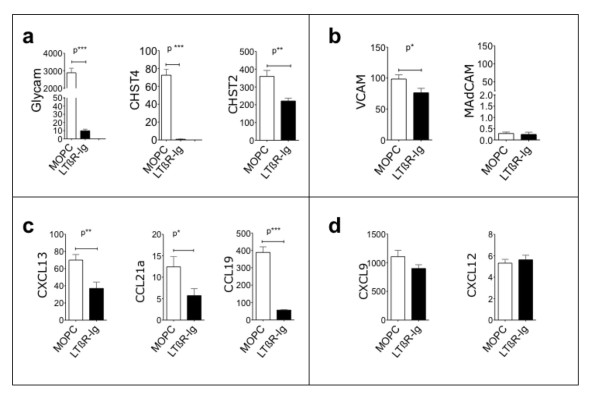
**Reduction of mRNA expression of HEV genes and chemokines by LTBR-Ig**. Real-time PCR analysis was performed on total mRNA isolated from lacrimal glands and lymph nodes from mice after treatment from 10 to 20 weeks with LTBR-Ig or MOPC-21. There were eight mice in each treatment group. The fold-change for each gene shown here was calculated relative to expression of that gene in healthy lacrimal glands from 20-week-old female NOD mice (*n *= 8). A comparison is shown of the effect of each treatment type on genes associated with HEV **(a)**; adhesion molecules VCAM and MAdCAM **(b)**; homeostatic chemokines **(c)**; and other chemokines **(d)**. The mean fold-change of eight samples and the standard deviation are plotted. Statistical analysis was by Student's T-test; P*< 0.05; P**< 0.01; P***< 0.001. HEV, high endothelial venule; LTBR-Ig; lymphotoxin-beta receptor-mouse immunoglobulin (Fc) chimeric inhibitor; MAdCAM, mucosal adressin/cell adhesion molecule; NOD, non-obese diabetic; PCR, polymerase chain reaction; VCAM, vascular cell adhesion molecule.

Significant numbers of CD11c-positive cells were present in lacrimal glands of the male 20-week-old NOD mice, as shown in Figure 2e. The percentage of CD11c-positive cells in lacrimal glands, expressed relative to all cells that were neither CD3-positive T-cells nor B220-positive B-cells, was reduced from a mean of 44% in lacrimal glands of MOPC-21 treated mice to only 15% after LTBR-Ig treatment, as shown in Figure 3e (*P *= 0.017).

The possibility that proliferation of B-cells in germinal center reactions might contribute to the large numbers of B-cells that accumulated in diseased lacrimal glands was examined. There was no indication of GL7 staining, a B-cell activation marker associated with germinal center (GC) reactions, either by FACS analyses of isolated lymphocytes or by immunohistochemical staining of lacrimal gland tissue sections (data not shown). There also was no evidence of peanut agglutinin (PNA) staining in lacrimal glands (data not shown). Finally, the rate of proliferation of T-cells and B-cells was determined by a 1 hour, *in vivo *pulse of bromodeoxyuridine (BrdU) followed by FACS analysis for BrdU incorporation. The analysis showed only a very low rate of proliferation (< 1%) in either T- or B-lymphocytes present in lacrimal glands and lymph nodes, while a rate of approximately 6% was seen in thymus for T-cells, as shown in Additional File 4. This low proliferation rate was not altered by the 8-week treatment of mice with LTBR-Ig.

### Lacrimal Gland Gene Expression Analysis

Lacrimal glands from mice that were untreated or treated with control protein or LTBR-Ig from weeks 8 to 16 were analyzed for differential gene expression. At week 16, using an arbitrary minimum of a 2-fold change, there were approximately 1,500 differentially expressed genes after LTBR-Ig treatment when compared to either the untreated or control protein cohorts. Genes down- or up-modulated by at least 5 fold are listed in Table 1 and Table 2. Among the most highly down modulated genes was *Glycam*, as expected, based on the ability of LTBR-Ig to affect the differentiation program that drives HEV addressin expression in secondary lymphoid organs [6]. The loss of L-selectin mRNA (*Sell*) expression is assumed to reflect the reduced entry of L-selectin positive lymphocytes into the gland via PNAd and HEV. Expression of a secreted phospholipase A2 (*Pla2g2d*) was also reduced 10-fold; this enzyme is selectively produced in lymphoid tissue and was previously shown to be under LTBR control [35,36]. Reflecting the reduced size of the leukocyte infiltrate, the expression of a large number of B-cell related genes as defined using the Immunological Genome Project database was reduced 5-fold or more by LTBR-Ig treatment (*Igh1 *(IgG2a heavy chain), *Cxcr5*, *Ebf1*, *Hhex, Fcrl1, Fcer2a, Btk, Cd37, Btla, B3gnt5, Cr2, Bank1, Cd38, H2-Ob *and *Ms4a1*. Genes that were increased by more than 80-fold included salivary protein 2 (*Spt2*), renin 1 (*Ren1*) and cysteine-rich secretory protein 3 (*Crisp3*). Additionally, 13 different kallikrein genes were highly upregulated along with mucin 10 (*Muc10*) and nerve growth factor-beta (*Ngfb*). The increased expression of various secretory proteins suggests that LTBR-Ig triggered increased glandular function and/or repair. While some of the most regulated genes were differentially expressed after only 4 weeks of treatment, the number of significant changes was much reduced compared to the number after 8-weeks of treatment (data not shown).

**Table 1:** Lacrimal gland gene expression reduced by treatment of mice with LTBR-Ig compared to control.

	Gene	Fold Change Down
Glycam1	glycosylation dependent cell adhesion molecule 1	29.02
Serpina1b	serine (or cysteine) preptidase inhibitor, clade A, member 1b	12.30
Sell	selectin, lymphocyte	11.43
Pla2g2d	phospholipase A2, group IID	10.53
Igh-1a	IgG2a heavy chain	8.47
Faim3	Fas apoptotic inhibitory molecule 3	8.09
Ccl19	CCL19 chemokine	7.67
Cxcr5	C-X-C Chemokine receptor type 5	7.59
Rnase6	ribonuclease, RNase A family, 6	7.19
Clca2	chloride channel calcium activated 2	6.77
Cxcr4	chemokine (C-X-C motif) receptor 4	6.71
Aff3	AF4/FMR2 family, member 3	6.46
Ebf1	early B-cell factor 1	6.42
Rasal3	RAS protein activator like 3	6.34
Hhex	hematopoietically expressed homeobox	6.23
Fcrl1	Fc receptor-like 1	6.18
Fcer2a	Fc receptor, IgE, low affinity II, alpha polypeptide	6.17
Btk	Bruton agammaglobulinemia tyrosine kinase	6.12
Cd37	CD37 antigen	6.09
Btla	B and T lymphocyte associated	6.07
B3gnt5	UDP-GlcNAc:betaGal beta-1,3-N-acetylglucosaminyltransferase 5	5.67
Cxcl13	chemokine (C-X-C motif) ligand 13	5.57
Fcrl1	Fc receptor-like 1	5.57
Ubd	ubiquitin D	5.54
Fam55c	family with sequence similarity 55, member C	5.53
Cr2	complement receptor 2	5.49
Fcer2a	Fc receptor, IgE, low affinity II, alpha polypeptide	5.39
H2-Ob	histocompatibility 2, O region beta locus	5.38
Bank1	B-cell scaffold protein with ankyrin repeats 1	5.36
Stat4	signal transducer and activator of transcription 4	5.26
Ptprc	protein tyrosine phosphatase, receptor type, C	5.25
Cd38	CD38 antigen	5.19
Ms4a1	membrane-spanning 4-domains, subfamily A, member 1	5.19
Pde7a	phosphodiesterase 7A	5.16
Lrrk2	leucine-rich repeat kinase 2	5.15
Plekha2	pleckstrin homology domain-containing, family A	5.14
Slamf6	SLAM family member 6	5.07
Slc2a3	solute carrier family 2 (facilitated glucose transporter), member 3	5.04
Tcrb-V13	T-cell receptor beta, variable 13	5.00

**Table 2:** Lacrimal gland gene expression elevated by treatment of mice with LTBR-Ig compared to control

	Gene	Fold Change Up
Spt2	salivary protein 2	116.39
Ren1	renin 1 structural	108.03
Crisp3	cysteine-rich secretory protein 3	88.55
Klk1b26	kallikrein 1-related petidase b26	83.06
Klk1b16	kallikrein 1-related peptidase b16	71.72
Abpb	androgen binding protein beta	51.14
Klk1b5	kallikrein 1-related peptidase b5	43.58
Klk1b9	kallikrein 1-related peptidase b9	42.18
Klk1b4	kallikrein 1-related pepidase b4	31.05
Klk1b8	kallikrein 1-related peptidase b8	30.37
Klk1b3	kallikrein 1-related peptidase b3	30.26
Muc10	mucin 10, submandibular gland salivary mucin	26.56
Egfbp2	epidermal growth factor binding protein type B	23.63
Klk1b4	kallikrein 1-related pepidase b4	18.95
Klk1b1	kallikrein 1-related peptidase b1	18.63
Smr2	submaxillary gland androgen regulated protein 2	15.83
Klk1b27	kallikrein 1-related peptidase b27	15.45
Klk1b21	kallikrein 1-related peptidase b21	14.58
Abpa	androgen binding protein alpha	9.94
Ngfb	nerve growth factor, beta	9.06
Klk1b24	kallikrein 1-related peptidase b24	8.59
Egf	epidermal growth factor	5.92
Klk1b11	kallikrein 1-related peptidase b11	5.29

Based on the Affymetrix gene-expression analyses we examined a few genes by quantitative PCR, as shown in Figure 3, including the addressin scaffold *Glycam1 *[37] and two sulfotransferase enzymes (*Chst2 *and *Chst4*) important in carbohydrate modifications of the adhesion molecule PNAd [38]. Two additional adhesion-molecules were examined as well, VCAM and MAdCAM. To avoid introducing confounding parameters due to use of a different mouse strain or a different age/developmental stage in the NOD strain, the mRNA level for each gene in the diseased lacrimal glands of the 20-week old male mice was compared to the level of expression of the same gene in the healthy lacrimal glands from female NOD mice of the same age. Histologic analysis of lacrimal glands from 14 to 20-week old female mice revealed only occasional indications of disease, such as small leukocyte infiltrates, and small B-cell clusters shown in Additional File 5. The mRNA expression of all genes we examined was higher in diseased lacrimal glands of male NOD mice compared to healthy lacrimal glands of female mice, as shown in Figure 3, and some genes were extremely highly induced such as GlyCAM (approximately 3,000-fold) and CXCL9 (approximately 1,000-fold).

While the expression of Glycam1, Chst4, CCL19 was greatly reduced by LTBR-Ig treatment (Figure 3a and 3c), expression of Chst2, CXCL13 and CCL21a were only modestly decreased (approximately 2-fold) when compared to the levels in MOPC-21 treated mice (Figure 3a and 3c). Expression of MAdCAM, CXCL9 and CXCL12 was not significantly altered (Figure 3b and 3d). MAdCAM addressin expression is often under LTBR control, but in this ectopic follicle setting, MAdCAM was not induced by disease.

### LTBR-Ig reduced CXCL13 protein in lacrimal glands

**Figure 4:**
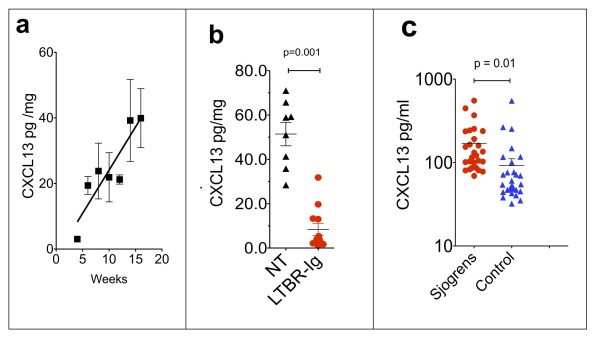
**CXCL13 protein measured by ELISA in mouse lacrimal glands and in sera from Sjögren's patients**. Homogenates made from individual lacrimal glands (*n *= 8) were analyzed for CXCL13 protein levels by ELISA **(a,b)**. CXCL13 protein increased with age/disease progression in lacrimal glands from untreated male NOD mice (a). LTBR-Ig treatment from 8 to 16 weeks reduced CXCL13 content of mouse lacrimal glands (b). Mean concentration of CXCL13 was significantly elevated in serum samples of Sjögren's syndrome patients (*n *= 27) versus healthy control sera (*n *= 30) **(c)**. The mean age of Sjögren's syndrome patients was 57.17 +/- 10.10 years, and for controls was 57.55 +/- 21.17 years. There was one man in each group. ELISA, enzyme linked immunosorbant assay; NOD, non-obese diabetic.

CXCL13 protein in lacrimal glands increased with age of the mice, and roughly mirrored the disease progression, as shown in Figure 4a. Interestingly, CXCL13 protein was very abundant in highly diseased lacrimal glands and the amount of CXCL13 per mg tissue equaled and sometimes exceeded the amount in cervical lymph nodes (approximately 40 pg/mg; data not shown).

As shown in Figure 4b, LTBR-Ig treatment from 8 to 16 weeks reduced the CXCL13 protein content of lacrimal glands approximately 5-fold compared to untreated control mice, from a mean of approximately 50 pg/mg to approximately 10 pg/mg tissue (*P *< 0.001). The reduction of mRNA and protein level of CXCL13 in lacrimal glands by LTBR-Ig is consistent with the approximately 5-fold reduction of B-cells present in lacrimal glands of LTBR-Ig treated mice, compared to control mice (Figure 2g). It is noteworthy that the amount of CXCL13 protein present in the diseased salivary glands of female NOD mice was approximately 10-times less than that of diseased lacrimal glands (data not shown).

### CXCL13 is elevated in sera of Sjögren's syndrome patients

The CXCL13 concentration in Sjögren's patient sera was measured by ELISA. The mean of the concentration of CXCL13 in sera from 27 patients diagnosed with primary Sjögren's syndrome was significantly higher (170 +/- 23 pg/ml) than that determined for thirty healthy control sera (92.0 +/- 18.9 pg/ml), as shown in Figure 4c, (*P *= 0.01). Examination of an independent cohort of sera from 18 Sjögren's syndrome patients gave comparable results (J. Browning, data not shown). Although it is known that immunoreactive CXCL13 is present in salivary glands of patients with Sjögren's syndrome [10], to our knowledge the amount of CXCL13 in the sera of patients has not yet been reported [1].

### LTBR-Ig reduced HEV in lacrimals

**Figure 5:**
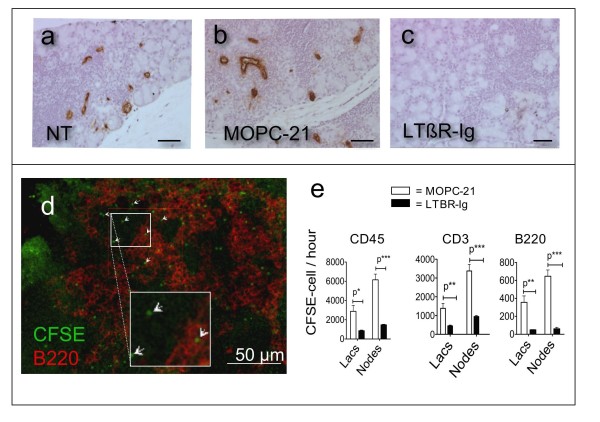
**LTBR-antagonism from 8 to16 weeks reduced the number of HEV in lacrimal glands and the uptake of CFSE-labeled lymphocytes into lacrimal glands**. Immuno-peroxidase visualized HEV (brown) after immunostaining with MECA-79 monoclonal antibody against PNAd in paraffin sections of lacrimal glands (NT = no treatment) **(a-c)**. The bar in (a-c) indicates 100 μm. Fluorescence photomicrograph of a lacrimal gland with B-cells (anti-B220, red) and CFSE-labeled cells indicated by arrowheads (CFSE, green) **(d)**. Quantification by FACS-analysis of CFSE-labeled lymphocytes among all lymphocytes isolated from lacrimal glands or cervical lymph nodes collected from mice (*n *= 4) that had been treated for 8 weeks with MOPC-21 (open bars) or LTBR-Ig (black bars). Qualitative assessment of ablation of MECA-79 stained HEV by LTBR-Ig treatments was performed more than five times. Quantitation of CFSE cell uptake was performed twice. CFSE, carboxyfluorescein succinimidyl ester; FACS, flow cytometry; HEV, high endothelial venule; LTBR, lymphotoxin-beta receptor; LTBR-Ig; lymphotoxin-beta receptor-mouse immunoglobulin (Fc) chimeric inhibitor.

HEV begin to appear in lacrimal glands of male NOD mice at about 8 weeks of age [22]. Since LTBR blockade reduced the numbers of HEV in lymph nodes and in diseased salivary glands of female NOD mice [6,23,26], the effect of LTBR-Ig treatment on the numbers of HEV in lacrimal glands was examined. Functional HEV react with monoclonal antibody MECA-79 as shown in Figure 5 (a to c). As illustrated in Figure 5a and 5b, HEV were abundant at 16 weeks of age in the lacrimal glands of untreated mice and mice treated with MOPC-21, but HEV were virtually absent from the lacrimal glands of mice treated with LTBR-Ig, shown in Figure 5c. The HEV content of lacrimal glands was quantified and expressed as a percent of the total area of each lacrimal gland. The HEV area was reduced approximately 25-fold by LTBR antagonism (8 to 16 weeks) to 0.03% (±0.01) compared to 1.05% (±0.19) for untreated mice and 0.75% (±0.19) for MOPC-21 treated mice. Similar results were obtained with both the prophylactic treatment regimen (8 to 16 weeks) and the therapeutic treatment regimen (14 to 22 weeks), as shown in Additional File 2b.

### LTBR-Ig reduced uptake rate of lymphocytes into lacrimal glands

To determine if the reduction of HEV content of lacrimal glands had functional consequences the rate of lymphocyte uptake into glands of control and LTBR-Ig treated mice was directly measured. CFSE-labeled lymphocytes were injected intravenously into recipient mice that had been treated with either MOPC-21 or LTBR-Ig from 8 to 16 weeks of age, and the number of CFSE-labeled cells present in the lacrimal glands following a 20-hour interval was determined by FACS analysis. A fluorescence-photomicrograph of the appearance of CFSE-labeled lymphocytes in lacrimal glands of a representative LTBR-Ig treated mouse is shown in Figure 5d in which CFSE-labeled cells appear green (examples are indicated by arrows) and B-lymphocytes are indicated in red (anti-B220-APC).

For quantitation of CFSE-labeled cells by flow-cytometry, leukocytes were isolated from lacrimal glands and then stained with a multicolor antibody cocktail containing anti-CD45, anti-CD4, anti-B220 and anti-CD62L. Live CD45-positive cells were gated and the CFSE-labeled cells (FITC channel) were detected among cells isolated from each pair of lacrimal glands. The number of CFSE-cells/200,000 CD45-positive cells was calculated and plotted for each group. As shown in Figure 5e, the uptake rate in mice treated with LTBR-Ig was reduced approximately three-fold for total CD45 positive cells, approximately four-fold for T-cells and approximately seven-fold for B-cells.

For comparative purposes we also measured CFSE-cell uptake by the cervical lymph nodes. Uptake of CFSE-labeled lymphocytes by lymph nodes occurred approximately two times faster than uptake by lacrimal glands, perhaps because lymph nodes have an intrinsically greater capacity for capturing naïve lymphocytes compared to the ectopic follicular-structures in diseased lacrimal glands. As previously reported [6], LTBR-Ig treatment also reduced the uptake of cells into lymph nodes, as shown in Figure 5e.

### LTBR-Ig Preserved Tear -flow Rate

**Figure 6:**
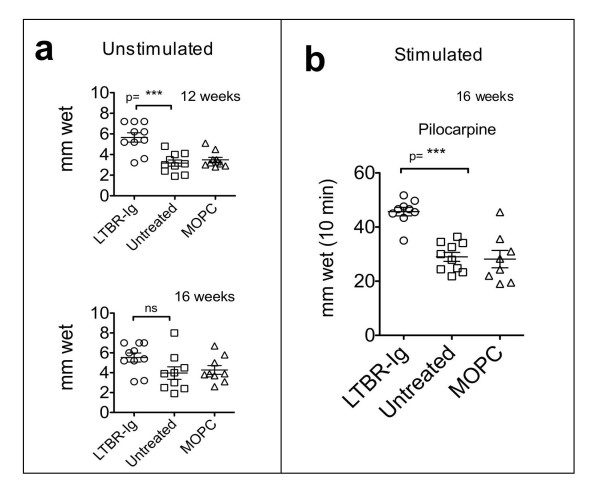
**LTBR-Ig enhanced the volume of tear-fluid secreted by lacrimal glands**. Mice were treated from age 8 to 16 weeks with LTBR-Ig (circles), with MOPC-21 control antibody (triangles), or were left untreated (squares). The amount of tear fluid secreted for each eye of each mouse was determined by insertion of a Zone-Quick phenol impregnated thread under the eyelid at the medial canthus. The length of thread wetted by capillary action in 30 seconds was measured and plotted **(a)**. The amount of tear fluid secreted was determined at age 12 weeks (after 4 weeks of treatment) and at 16 weeks of age (after 8 weeks of treatment), as indicated on each graph in (a). The rate of tear fluid secretion was also determined in the presence of pharmacologic stimulation. In a separate experiment, after 8 week treatments as indicated, each mouse was injected with pilocarpine to maximally stimulate tear fluid secretion, and after 5 minutes, the amount of tear fluid secreted by each eye during the next 10 minutes was measured by wetting of a Zone-Quick thread placed under the lid **(b)**. Statistical analysis was by the two-tailed Student's test. P*< 0.05; P**< 0.01; P*** < 0.001. This type of experiment was performed four times. LTBR-Ig; lymphotoxin-beta receptor-mouse immunoglobulin (Fc) chimeric inhibitor.

To assess whether the reduced lymphocytic infiltrates in lacrimal glands of LTBR-Ig-treated mice was associated with a reduction in loss of lacrimal gland function, two parameters related to ocular health were examined, the rate of tear fluid secretion and the integrity of the ocular surface. In previous studies in mice, the basal tear fluid secretion rate was measured for unstimulated lacrimal glands, as well as after stimulation of the lacrimal glands to their maximal secretion rate by the systemic administration of pilocarpine, a parasympathetic nervous system agonist. In this study, both methods were used, as shown in Figure 6a and 6b.

As shown in Figure 6a, the basal tear fluid secretion rate was significantly greater after LTBR-Ig treatment for 4 weeks compared to MOPC-21 treated control mice (*P *= 0.0002), but after 8 weeks of treatment the difference was not quite statistically significant (*P *= 0.056), although a protective trend was apparent.

To measure the pilocarpine stimulated, maximal tear secretion rate, excess tear fluid that had collected under the eyelid during the first 5 minutes after injection of pilocarpine was first removed, and then the amount of tear fluid that was secreted for the next 10 minutes was measured. As shown in Figure 6b, mice treated with LTBR-Ig for 8 weeks secreted more tear fluid than untreated control mice or control mice treated with MOPC-21 (*P *= 0.0001).

### LTBR-Ig preserved the integrity of the ocular surface

**Figure 7:**
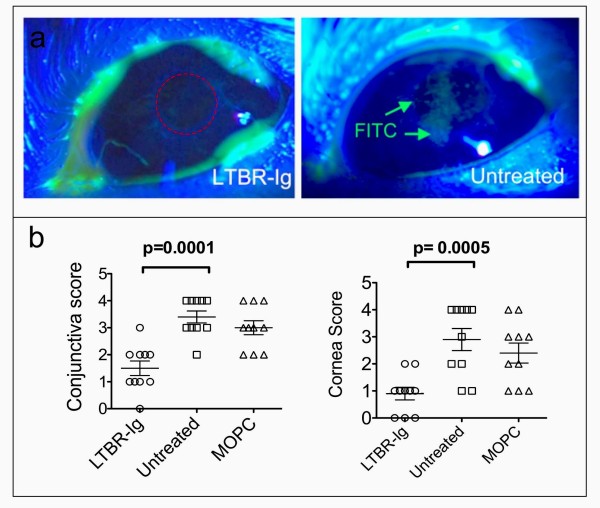
**LTBR-Ig treatment reduced the loss of epithelial integrity of conjunctiva and cornea**. Selected examples of the appearance of mouse eyes 5 minutes after applying a 5 μl drop of FITC-PBS **(a)**. Eyes of anesthetized mice were illuminated with Cobalt-blue filtered light and observed and photographed though a slit-lamp microscope (a). Note that the bright green outline of the eye is pooled excess FITC-PBS and does not indicate epithelial damage. An example of a corneal score of zero for an LTBR-Ig treated mouse (left) and a score of 4 for an untreated mouse (right) is shown, where the red circle in (a) indicates the region defined as the cornea. Mice were treated from age 8 to 16 weeks with LTBR-Ig (circles in **b**), were left untreated (squares in b), or were treated with MOPC-21 control antibody (triangles in b). The scores for the conjunctiva (left graph) and the cornea (right graph) for one eye of each mouse (contra-lateral to the eye used to measure tear secretion) were plotted and the mean score indicated (b). This experiment was performed twice. FITC, fluorescein isothyocyanate; LTBR-Ig; lymphotoxin-beta receptor-mouse immunoglobulin (Fc) chimeric inhibitor; PBS, phosphate-buffered saline.

The integrity of the ocular epithelial surface serves as an indirect measure of the long-term consequences of net changes in the protein composition of the tear film, in combination with the rate/volume of tear fluid secretion. Application of aqueous FITC is a simple and effective way to assess the condition of the ocular surface. In this analysis, perfectly intact epithelium does not bind FITC. In contrast, defects in the ocular surface epithelial cell layer allow FITC to access components of the underlying cell layers and extra-cellular matrix where the FITC then can adhere and is visible under UV-enriched illumination with a cobalt blue filter. Using an established, semi-quantitative scoring system, FITC staining of the corneal/conjunctival surface was evaluated by slit-lamp microscopic eye examination of each eye (*n *= 10), and the corneal and conjunctival areas were scored on a scale of 1 to 4 (shown in Additional File 6) by an observer unaware of the type of treatment given to each mouse. The cornea was defined as the region directly over the center of the eye and extending to the perimeter of the pupil (red circle, Figure 7a), and the conjunctiva was the remainder of the ocular surface. Selected examples of the extremes of the range of FITC staining are shown in Figure 7a, in which a zero score is illustrated with an LTBR-Ig-treated mouse and a maximal score of 4 is illustrated with an untreated mouse. As shown in Figure 7b, LTBR-Ig treatment of mice for 8 weeks resulted in significantly lower scores for FITC staining for both conjunctiva (*P *= 0.0001) and cornea (*P *= 0.0005), reflecting less damage to the ocular epithelial surface.

## Discussion

In this study, antagonism of the LTBR axis greatly reduced CXCL13 in diseased extra-orbital lacrimal glands and profoundly reduced the B-lymphocyte burden in the glands. These effects of LTBR antagonism on B-cells and CXCL13 are especially interesting in light of the recent recognition of the importance of B-cells in Sjögren's syndrome. Certain B-cell subsets found in the glands of Sjögren's patients, possibly due to aberrant expression of B-cell growth and differentiation factor BAFF, have been proposed to contribute to the pathogenesis in Sjögren's [39-41]. That B-cells contribute greatly to other autoimmune diseases has been verified clinically, as exemplified by the success of B-cell depletion therapy for treatment of rheumatoid arthritis [42]. Recently, a report of a double blind, randomized clinical trial of B-cell depletion by rituximab showed beneficial effects in Sjögren's patients suggesting that B-cell targeted therapies may be useful to treat Sjögren's syndrome as well[43].

The health of the ocular surface depends on diverse factors, including tear osmolarity, the protein/proteoglycan constituents of tear fluids, and the volume of tear fluid delivered to the ocular surface. Tear fluid contains over 400 protein constituents that undoubtedly play important, although currently poorly defined roles in corneal and conjunctiva epithelial integrity [44,45]. In the male NOD mouse, within the first 6 weeks of age lacrimal glands undergo markedly altered gene expression [19,20], including elevated expression of cathepsin, increased extra-cellular matrix degradation and altered lipid homeostasis [46,47]. These changes precede the massive accumulation of leukocytes that culminates in overt exocrinopathy by 12 to 16 weeks of age [48,49]. In this study, differential gene expression analysis with Affymetrix gene arrays showed that antagonism of the LTBR-axis altered the expression of a wide variety of genes related to lymphocyte trafficking, lymphocyte function and lacrimal gland function, and these changes coincided with improvement in tear fluid secretion and ocular integrity, two measures of overall ocular health. While the precise mechanism(s) of action are not yet clear, the net result of LTBR antagonism was a diminution of the loss of tear production by lacrimal glands and partial protection from loss of the integrity of the ocular surface.

The beneficial effects of LTBR antagonism reported here are likely linked to diverse factors. It is the combined effect of reduced tear fluid volume and the altered composition of tear fluid due to lacrimal gland disease that results in damage to the epithelial cell layer of the ocular surface. The reduced delivery of protective antibodies and of growth factors such as epidermal growth factor (EGF) and nerve growth factor (NGF), which have been implicated in corneal epithelial homeostasis, are thought to contribute to loss of ocular surface integrity [50]. Administration of LTBR-Ig spared some of these protein factors of tear fluid from disease-driven down regulation. For example, LTBR-Ig treatment diminished the disease associated losses in mRNA expression of NGF (approximately 9-fold higher than in control antibody treated mice), EGF (approximately 6-fold higher), mucin 10 (approximately 30-fold higher) and EGF-binding protein (approximately 24-fold higher) and may have helped to maintain the integrity of the ocular surface, as these proteins may have roles in corneal epithelial cell renewal (see Table 1). Increased repair of the ocular surface and the gland itself (that is, sustained growth factor expression) may be factors in the beneficial outcome of LTBR blockade. Future studies are planned to evaluate this possibility more directly.

The gene expression patterns we observed by Affymetrix chip assay clearly reflected the profound reduction in the number of leukocytes in lacrimal glands after antagonism of the LTBR axis for 8 weeks. Many genes related to B-cell signaling pathways that are elevated by disease progression were reduced by LTBR-Ig treatments (data not shown). The ability of LTBR-Ig to prevent and reverse the differentiation of venules near the leukocyte infiltrates to HEV was also reflected in the gene expression data, with *Glycam1 *and *Chst4 *being profoundly reduced, resulting in a co-ordinate reduction of L-selection expression, probably because of reduced entry of naïve lymphocytes into lacrimal glands. Uptake of fluorescent labeled lymphocytes into lacrimal glands was reduced several fold in mice treated with LTBR-Ig compared to controls, demonstrating functional consequences of the effects of LTBR-Ig treatment on HEV. Thus, a major action of LTBR antagonism was the reduction of the rate of entry of lymphocytes into lacrimal glands probably due to the virtual elimination of HEV that expressed PNAd.

The tight association between the presence of HEV and B-cell accumulation is consistent with the notion that HEV formation is an early event in TLT formation. However, in diseased lacrimal glands, the lymphoid cell aggregates appear suspended in an intermediate or immature state since they were slow to acquire the micro-architecture of mature TLT, such as well-segregated T-cell areas and B-cell areas with associated FDC networks. This immature state is very apparent when compared to submandibular glands in female NOD mice [26]. The factors that prevent or delay the organization of the lymphocyte aggregates in lacrimal glands are not understood. However, one striking difference was a paucity of lymphatic vessels near lymphoid aggregates of lacrimal glands compared to the TLT of diseased submandibular glands in female NOD mice. LYVE-1 immunoreactive lymphatic vessels were abundant near TLT in submandibular glands of female mice, but lymphatic vessels were essentially absent from the perimeter of the large B-cell aggregates in diseased lacrimal glands (data not shown). Lymphatic vessels have been reported to be relatively scarce in lacrimal glands, which may make any lymphangiogenic response in lacrimal glands less robust than in salivary glands[51]. Lymph angiogenesis is tightly linked to mature TLT development, as demonstrated in an experimental model of Hashimoto's disease in the thyroid where robust lymph angiogenesis was driven primarily by T-cells during development of ectopic follicles [23]. Interestingly, it is known that B-cells exit lymph nodes in part by directly entering lymphatic vessels in the cortex of lymph nodes [52]; a difficulty in exiting lacrimal glands via lymphatic vessels in a similar fashion could contribute to accumulation of B-cells. We speculate that high levels of CXCL13 and the lack of robust lymph angiogenesis in lacrimal glands contribute to limited egress of B-cells from glands.

A discordance was noted between the modest (approximately two-fold) down-modulation of CXCL13 mRNA and the approximately five-fold reduction of CXCL13 protein in lacrimal glands after 8 weeks of LTBR-Ig treatment. One possibility is that LTBR-Ig treatment affects elements on the extracellular matrix and peri-cellular matrix of HEV that retain CXCL13 in the microenvironment. Interestingly, lacrimal glands contained approximately 10 times more CXCL13/mg tissue measured by ELISA than did salivary glands of female NOD mice of comparable age (data not shown). This was particularly surprising as FDC networks, a known source of CXCL13 production [53], were abundant in salivary gland TLT [26] but were rare in lacrimal glands. The source(s) of the large amount of CXCL13 produced during the course of lacrimal gland disease remains under investigation. Early *in situ *hybridization studies in human salivary gland biopsies from Sjögren's patients have suggested that ductal epithelial cells may produce CXCL13 [54]. Reports have been made that monocytic cells [55] and CD11c dendritic cells [56,57] are potential sources of CXCL13, and we will investigate this possibility in future studies. Other possibilities include follicular-B helper T-cells which make CXCL13 in the follicles of secondary lymphoid tissues [53], as well as stromal organizer cells which may be present where TLT development is occurring [4,58,59]. Yet another possibility is the induction of CXCL13 expression in macrophages by biglycan, a byproduct of degradation of the extra-cellular matrix known to occur in diseased lacrimal glands [46,60].

## Conclusions

These data suggest that antagonism of LTBR may be effective as a therapy to treat the dry eye aspect of Sjögren's syndrome. The efficacy is likely due to the combined effects of the modulation of mRNA expression of a number of functional and disease associated genes in the lacrimal gland, a reduction of CXCL13 protein in lacrimal glands, virtual elimination of HEV in lacrimal-glands and reduced lymphocyte uptake by diseased lacrimal glands. Overall, LTBR antagonism produced beneficial effects on tear fluid secretion and the integrity of the ocular surface. These therapeutic effects were achieved at a stage of disease that preceded the full organization of the lymphocyte aggregates into functional tertiary lymphoid follicles, suggesting that mere reduction of the lymphocyte burden was sufficient to protect lacrimal gland function. Elucidation of the detailed mechanisms responsible for these beneficial effects awaits additional studies.

## Abbreviations

AID: activation-induced cytidine deaminase; BAFF: B-cell activating factor; BrdU: bromodeoxyuridine; CFSE: carboxyfluorescein succinimidyl ester; DAB: diamino-benzidine; EGF: epidermal growth factor; ELISA: enzyme linked immunosorbant assay; FACS: flow cytometry; FITC: fluorescein isothyocyanate; FDC: follicular dendritic cells; GC: germinal center; H & E: hematoxylin and eosin; HEV: high endothelial venule; HRP: horseradish peroxidase; ICAM: intercellular adhesion molecule; KS: keratoconjunctivitis sicca; LTBR: lymphotoxin-beta receptor; LTBR-Ig; lymphotoxin-beta receptor-mouse immunoglobulin (Fc) chimeric inhibitor; MAdCAM: mucosal adressin/cell adhesion molecule; MZB: marginal zone B-cell; NGF: nerve growth factor; NOD: non-obese diabetic; OCT: optimal cutting temperature embedding compound; PBS: phosphate-buffered saline; PCR: polymerase chain reaction; PNAd: peripheral lymph node addressin; RIPA: radioimmuno-precipitation assay; TLT: tertiary lymphoid tissue; VCAM: vascular cell adhesion molecule.

## Competing interests

JB, AP and JLB are employees of Biogen Idec engaged in development of LTBR-Ig for clinical use. The remaining authors have no competing interests.

## Authors' contributions

RAF directed the entire project with contributions from AIB, MG, KS, JAK and JLB. RAF, SMK, SGW, JLB and AP performed FACS analyses, Q-PCR, histology, slit-lamp microscopy, ELISA and other animal work. JB performed Affymetrix gene analyses. CM performed CXCL13 assays on patient sera. All authors read and approved the final manuscript.

## Supplementary Material

Additional File 1 **Histology and fluorescence imaging of diseased lacrimal-glands**. H+E stained paraffin tissue section of a large leukocyte aggregate in the lacrimal-gland of a 16-week- old male NOD mouse (a). Overlay of fluorescence photomicrographs of a frozen section of a lacrimal gland from a 52-week-old male NOD mouse, after immunostaining with anti-CD3 (red), anti-B220 (blue) and anti CD21/35 (green) (b). Note that highly developed and tightly clustered T-cell areas are present (red) and an example is shown in the inset of a small FDC-network stained by anti-CD21/35 (green). Bar equals 100 μm. Click here for file

Additional File 2 **Delayed LTBR-Ig treatment (14 to 22 weeks) reduced infiltrates and HEV in lacrimal glands**. Photomicrographs of paraffin tissue sections of lacrimal glands stained with H&E (a, upper) to show leukocyte aggregates or by monoclonal antibody MECA-79, immuno-peroxidase and DAB substrate (brown) to show HEV (a, lower). Note representative images of the appearance of lacrimal glands at the start of treatment (14 weeks) and after treatment (22 weeks) with MOPC-21 (a, upper right) or LTBR-Ig (a, lower right). Quantification of the percent of the total area of lacrimal-gland examined that is occupied by HEV (top graph) and the number/unit area of HEV (lower graph) with the effect of treatment with MOPC or LTBR-Ig versus untreated mice is indicated on the graph. The mean of 20 measurements taken from tissue sections from five mice is plotted with the standard deviation. Note the 5-fold reduction in HEV content by LTBR-Ig treatment. Click here for file

Additional File 3 **FACS analysis plots and gating for MZB of lymphocytes from spleen and lacrimal glands**. Representative plots are shown for cells isolated from spleen (left panel) and from lacrimal glands (right panel) as indicated, after staining for B220, CD4, CD8, CD21 and CD23. Plots shown for cells from each organ are (counterclockwise): forward and side scatter (upper left), ungated cells B220 and CD4+CD8 combined (lower left), gated first on live lymphocytes then B220 and CD4+CD8 combined (lower right), gated on B220-positive cells then CD23 and CD21 (upper right). Gates for marginal zone B-cells (MZB) and follicular B-cells (FB) are indicated. Note that MZB are much more abundant in spleen (approximately 17%) than in lacrimal glands (approximately 3%) in the representative analyses shown. The vast majority of B-cells in either organ are follicular B-cells, for spleen approximately 73% and for lacrimal glands approximately 79%. Mice were treated from 8 to 16 weeks of age. Click here for file

Additional File 4 **Cell proliferation assessed by BrdU incorporation in thymus, lymph nodes and lacrimal glands**. Mice were treated from 8 to 16 weeks with LTBR-Ig (*n *= 4) or MOPC-21 (*n *= 4) then BrdU was injected one hour before euthanasia. Lymphocytes were isolated from thymus, pooled cervical lymph nodes and lacrimal glands, and were stained with fluorescent-conjugated ant-iCD45, anti-BrdU, anti-B220, and anti-CD3 and then analyzed by flow cytometry. The percentage of T-cells (red columns) and B-cells (blue columns) that had incorporated BrdU were plotted for thymus (left), cervical lymph nodes (center) and lacrimal glands (right). Click here for file

Additional File 5 **Comparison of female and male lacrimal-gland B-cell infiltrates and HEV content**. Lacrimal glands were collected from 17 week old male and female mice as indicated (*n *= 5) and frozen tissue sections were stained with anti-B220 for B-cells (top) and adjacent tissue sections were stained with MECA-79 for PNAd expressed on HEV (bottom) by immunoperoxidase and DAB substrate development (brown). Arrows (top left) indicate two representative, small, B-cell aggregates observed in lacrimal glands from female mice. Lower micrographs are higher magnification images of the same areas marked by black rectangles in upper micrographs, on tissue sections stained with MECA-79 to visualize PNAd-positive HEV. Arrow in lower left image marks the only HEV observed associated with the small B-cell aggregate in the representative lacrimal gland from a female mouse. Click here for file

Additional File 6 **Slit-lamp microscope images of examples of FITC staining of eyes of mice**. Mice were lightly anesthetized and a drop of aqueous FITC placed onto the ocular surface and the eyelids manually closed to spread FITC over the entire surface. Mice awakened to blink naturally, and were anesthetized again after 5 minutes for examination of the entire ocular surface by slit-lamp microscopy. Photographs were taken through a slit-lamp microscope using Cobalt-blue filtered light. Representative examples of each score given (0-4) are shown as indicated on the graph. A score of 0 was for no damage visible and a score of 4 was given for severe damage to the epithelium, reflected by the amount of FITC staining of the eye surface. Note that the very bright green at the perimeter of the eye is pooled, excess FITC and does not indicate ocular damage. Click here for file
