# Supplementary figures and images for: Lymphotoxin-beta receptor blockade reduces CXCL13 in lacrimal glands and improves corneal integrity in the NOD model of Sjögren's syndrome
Source: Arthritis Res Ther. 2011 Nov 1;13(6):R182. doi: 10.1186/ar3507 (PMC3334628; doi:10.1186/ar3507)

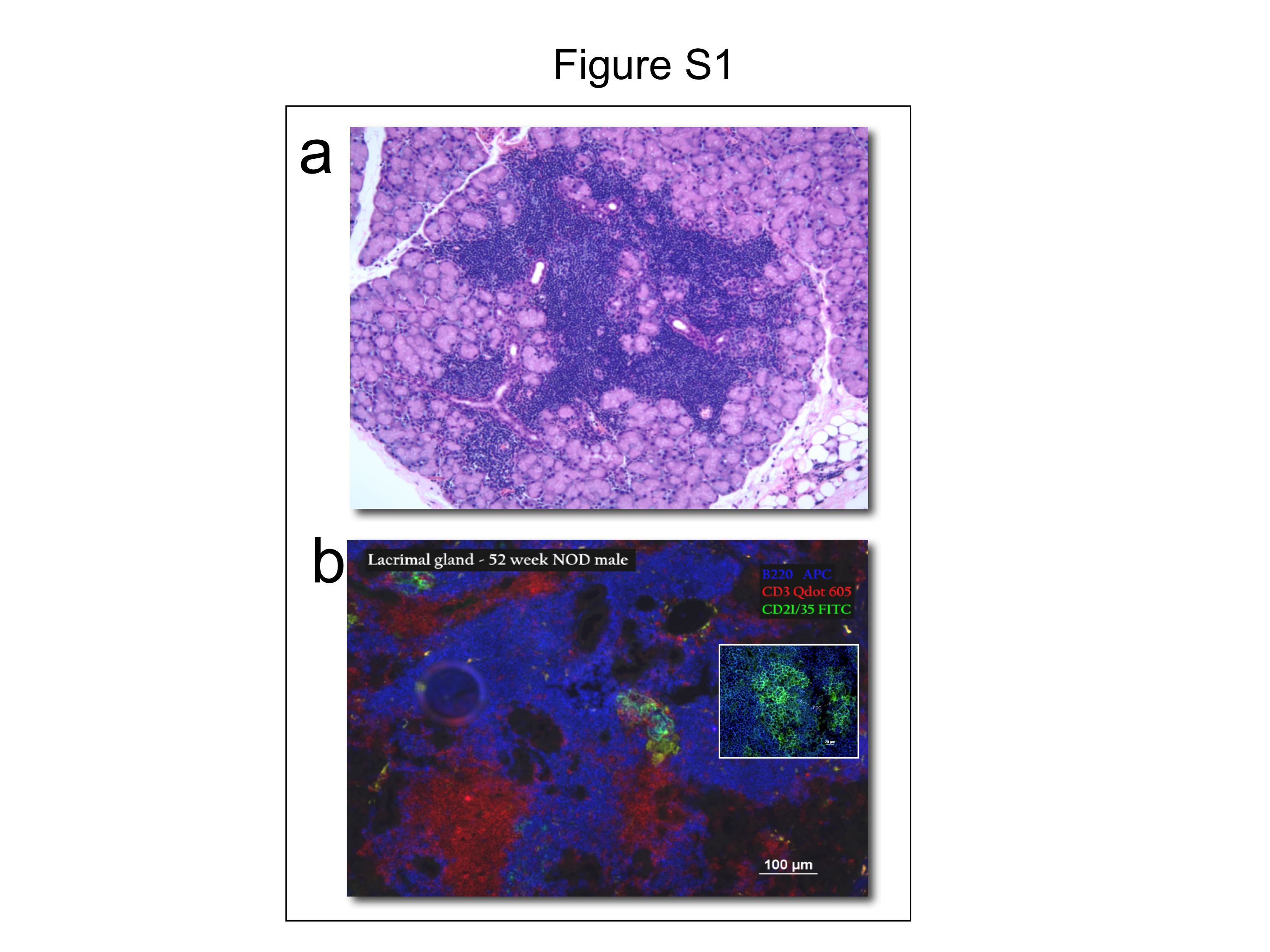

Supplement: Additional File 1 — Histology and fluorescence imaging of diseased lacrimal-glands. H+E stained paraffin tissue section of a large leukocyte aggregate in the lacrimal-gland of a 16-week- old male NOD mouse (a). Overlay of fluorescence photomicrographs of a frozen section of a lacrimal gland from a 52-week-old male NOD mouse, after immunostaining with anti-CD3 (red), anti-B220 (blue) and anti CD21/35 (green) (b). Note that highly developed and tightly clustered T-cell areas are present (red) and an example is shown in the inset of a small FDC-network stained by anti-CD21/35 (green). Bar equals 100 μm. [file ar3507-S1.JPEG]

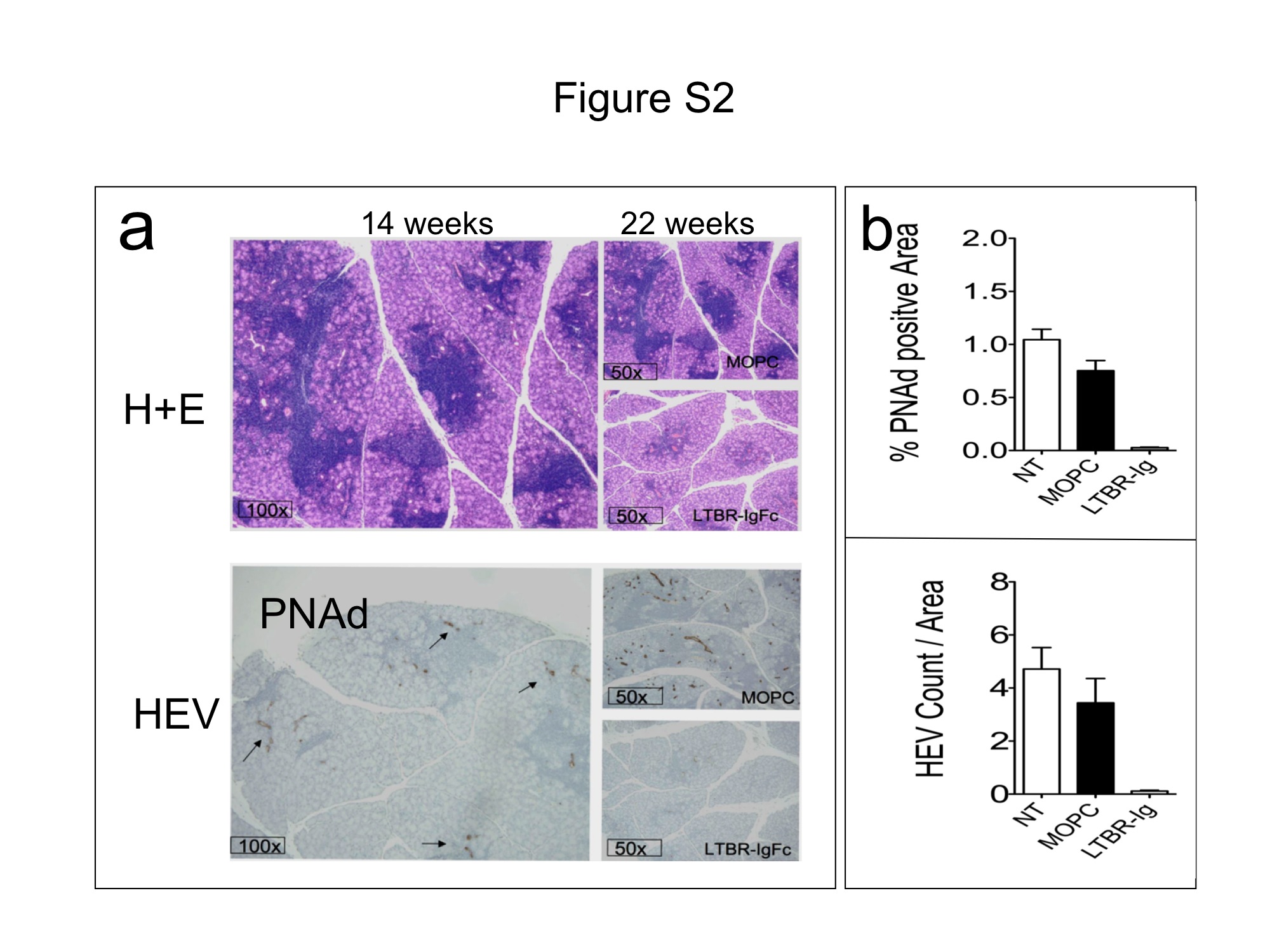

Supplement: Additional File 2 — Delayed LTBR-Ig treatment (14 to 22 weeks) reduced infiltrates and HEV in lacrimal glands. Photomicrographs of paraffin tissue sections of lacrimal glands stained with H&E (a, upper) to show leukocyte aggregates or by monoclonal antibody MECA-79, immuno-peroxidase and DAB substrate (brown) to show HEV (a, lower). Note representative images of the appearance of lacrimal glands at the start of treatment (14 weeks) and after treatment (22 weeks) with MOPC-21 (a, upper right) or LTBR-Ig (a, lower right). Quantification of the percent of the total area of lacrimal-gland examined that is occupied by HEV (top graph) and the number/unit area of HEV (lower graph) with the effect of treatment with MOPC or LTBR-Ig versus untreated mice is indicated on the graph. The mean of 20 measurements taken from tissue sections from five mice is plotted with the standard deviation. Note the 5-fold reduction in HEV content by LTBR-Ig treatment. [file ar3507-S2.JPEG]

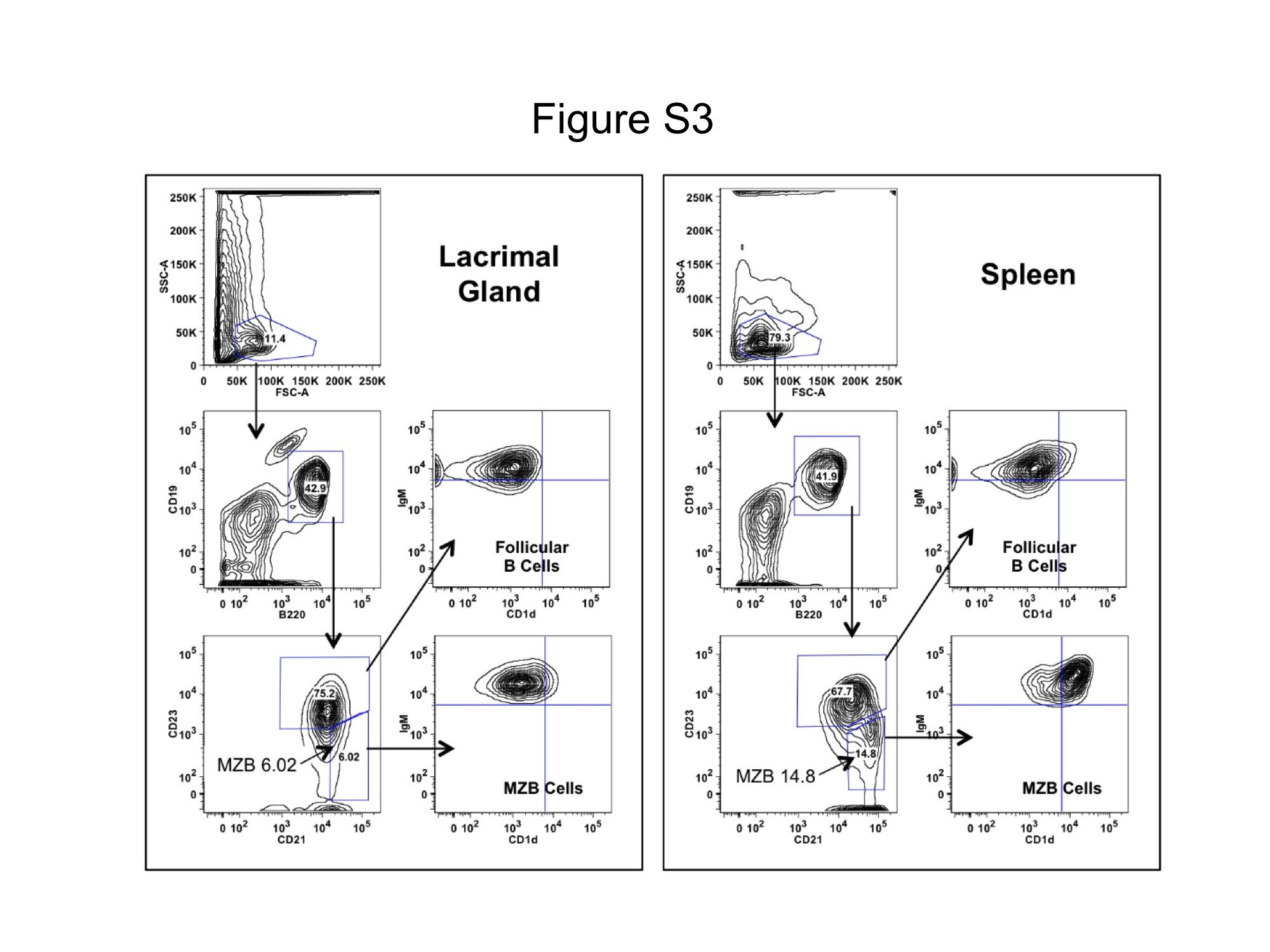

Supplement: Additional File 3 — FACS analysis plots and gating for MZB of lymphocytes from spleen and lacrimal glands. Representative plots are shown for cells isolated from spleen (left panel) and from lacrimal glands (right panel) as indicated, after staining for B220, CD4, CD8, CD21 and CD23. Plots shown for cells from each organ are (counterclockwise): forward and side scatter (upper left), ungated cells B220 and CD4+CD8 combined (lower left), gated first on live lymphocytes then B220 and CD4+CD8 combined (lower right), gated on B220-positive cells then CD23 and CD21 (upper right). Gates for marginal zone B-cells (MZB) and follicular B-cells (FB) are indicated. Note that MZB are much more abundant in spleen (approximately 17%) than in lacrimal glands (approximately 3%) in the representative analyses shown. The vast majority of B-cells in either organ are follicular B-cells, for spleen approximately 73% and for lacrimal glands approximately 79%. Mice were treated from 8 to 16 weeks of age. [file ar3507-S3.JPEG]

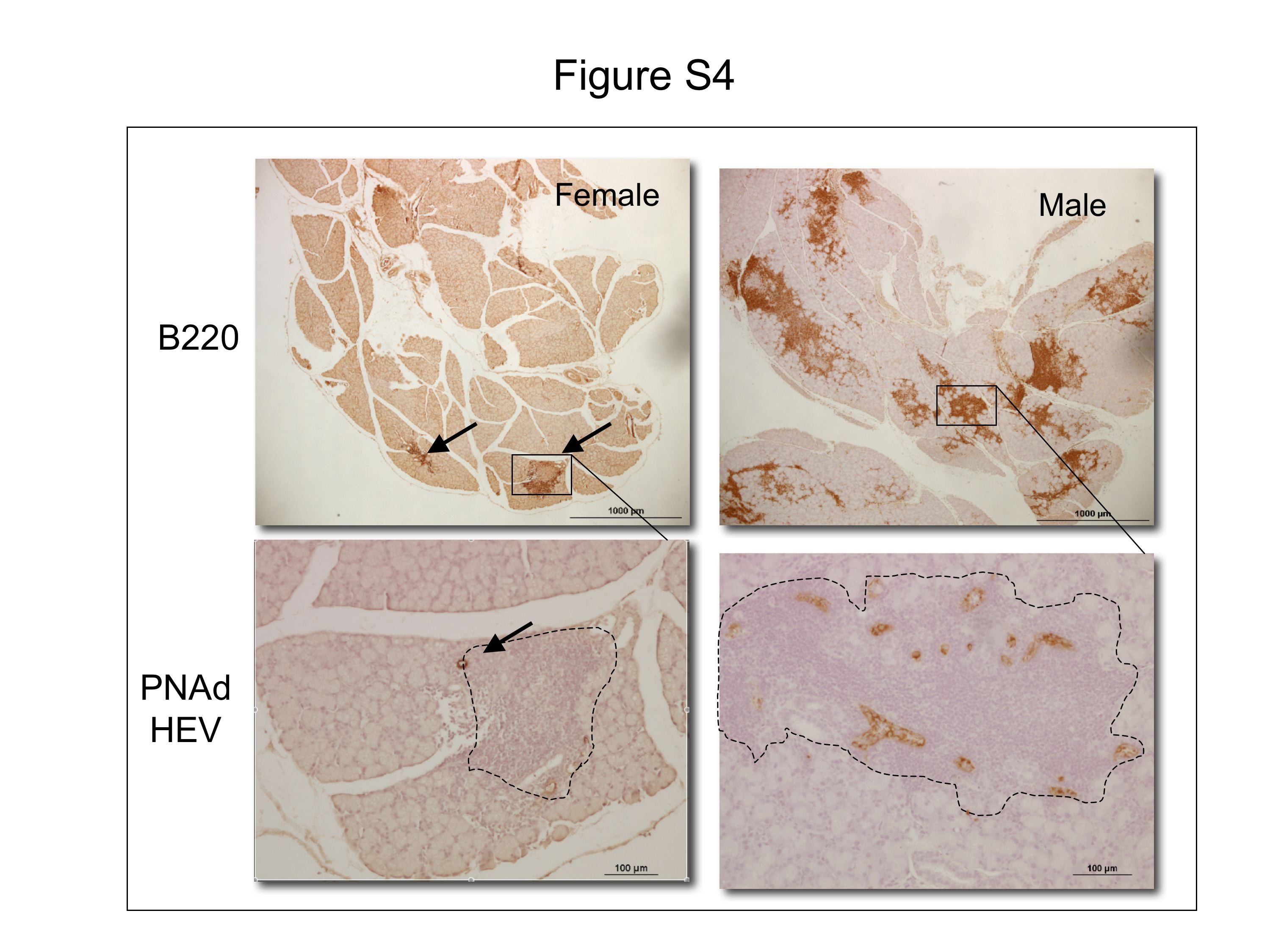

Supplement: Additional File 4 — Cell proliferation assessed by BrdU incorporation in thymus, lymph nodes and lacrimal glands. Mice were treated from 8 to 16 weeks with LTBR-Ig (n = 4) or MOPC-21 (n = 4) then BrdU was injected one hour before euthanasia. Lymphocytes were isolated from thymus, pooled cervical lymph nodes and lacrimal glands, and were stained with fluorescent-conjugated ant-iCD45, anti-BrdU, anti-B220, and anti-CD3 and then analyzed by flow cytometry. The percentage of T-cells (red columns) and B-cells (blue columns) that had incorporated BrdU were plotted for thymus (left), cervical lymph nodes (center) and lacrimal glands (right). [file ar3507-S4.JPEG]

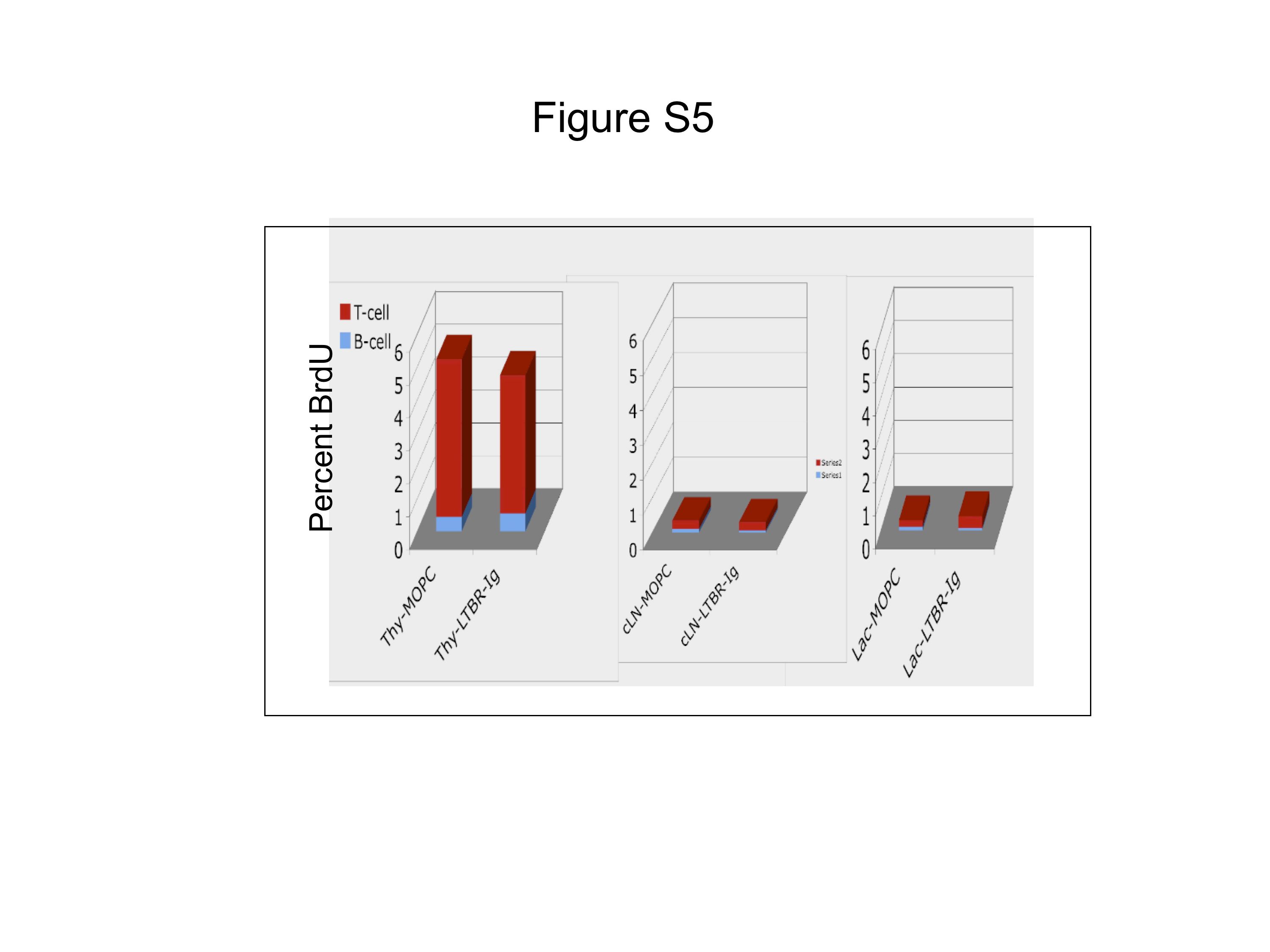

Supplement: Additional File 5 — Comparison of female and male lacrimal-gland B-cell infiltrates and HEV content. Lacrimal glands were collected from 17 week old male and female mice as indicated (n = 5) and frozen tissue sections were stained with anti-B220 for B-cells (top) and adjacent tissue sections were stained with MECA-79 for PNAd expressed on HEV (bottom) by immunoperoxidase and DAB substrate development (brown). Arrows (top left) indicate two representative, small, B-cell aggregates observed in lacrimal glands from female mice. Lower micrographs are higher magnification images of the same areas marked by black rectangles in upper micrographs, on tissue sections stained with MECA-79 to visualize PNAd-positive HEV. Arrow in lower left image marks the only HEV observed associated with the small B-cell aggregate in the representative lacrimal gland from a female mouse. [file ar3507-S5.JPEG]

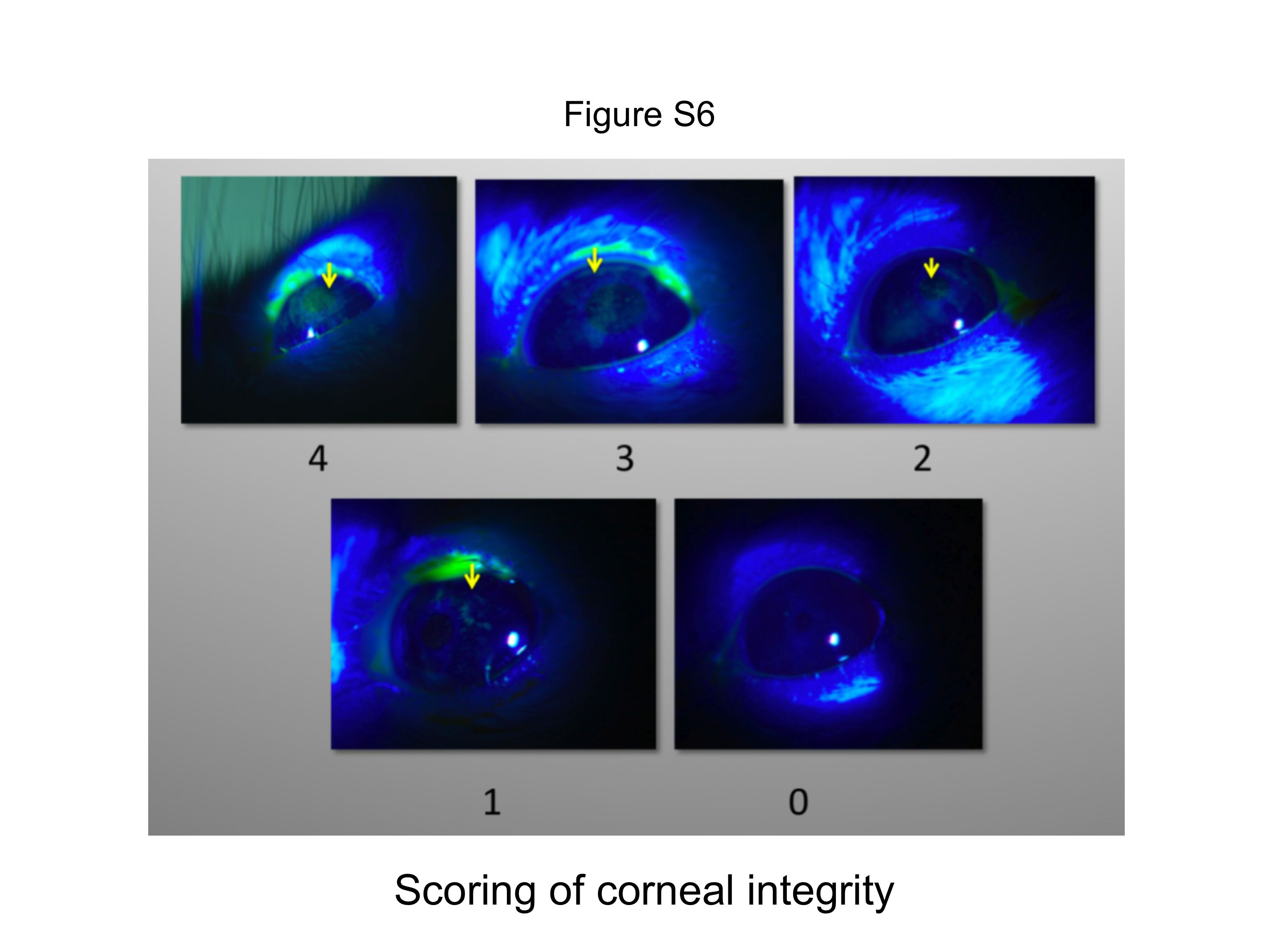

Supplement: Additional File 6 — Slit-lamp microscope images of examples of FITC staining of eyes of mice. Mice were lightly anesthetized and a drop of aqueous FITC placed onto the ocular surface and the eyelids manually closed to spread FITC over the entire surface. Mice awakened to blink naturally, and were anesthetized again after 5 minutes for examination of the entire ocular surface by slit-lamp microscopy. Photographs were taken through a slit-lamp microscope using Cobalt-blue filtered light. Representative examples of each score given (0-4) are shown as indicated on the graph. A score of 0 was for no damage visible and a score of 4 was given for severe damage to the epithelium, reflected by the amount of FITC staining of the eye surface. Note that the very bright green at the perimeter of the eye is pooled, excess FITC and does not indicate ocular damage. [file ar3507-S6.JPEG]
